# Increased quadriceps muscle strength after medial meniscus posterior root repair is associated with decreased medial meniscus extrusion progression

**DOI:** 10.1186/s12891-023-06858-0

**Published:** 2023-09-12

**Authors:** Koki Kawada, Takayuki Furumatsu, Mikao Fukuba, Masanori Tamura, Naohiro Higashihara, Yuki Okazaki, Yusuke Yokoyama, Yoshimi Katayama, Masanori Hamada, Toshifumi Ozaki

**Affiliations:** Department of Orthopaedic Surgery, Okayama University Graduate School of Medicine, Dentistry, and Pharmaceutical Sciences, 2‑5‑1 Shikata‑Cho, Kita‑Ku, Okayama 700‑8558 Japan

**Keywords:** Clinical score, Medial meniscus, Medial meniscus extrusion, Muscle strength, Posterior root tear, Quadriceps

## Abstract

**Background:**

This study aimed to assess quadriceps muscle strength after medial meniscus (MM) posterior root repair and determine its relationship with clinical scores and MM extrusion (MME).

**Methods:**

Thirty patients who underwent pullout repair for MM posterior root tear and were evaluated for quadriceps muscle strength preoperatively and at 1 year postoperatively were included in this study. Quadriceps muscle strength was measured using the Locomo Scan-II instrument (ALCARE, Tokyo, Japan). MME and clinical scores (i.e., Knee Injury and Osteoarthritis Outcome Score [KOOS], International Knee Documentation Committee score, Lysholm score, Tegner score, and visual analog scale pain score) were evaluated preoperatively and at 1 year postoperatively, and second-look arthroscopy was performed at 1 year postoperatively. Wilcoxon's signed-rank test was used to compare each measure pre- and postoperatively. Pearson's correlation coefficient was used to assess the correlation with quadriceps muscle strength values. Multiple regression analysis was performed to identify factors associated with the change in MME (ΔMME).

**Results:**

Second-look arthroscopy confirmed continuity of the posterior root in all patients. The quadriceps muscle strength measured at 1 year postoperatively (355.1 ± 116.2 N) indicated significant improvement relative to the quadriceps muscle strength measured preoperatively (271.9 ± 97.4 N, *p* < 0.001). The MME at 1 year postoperatively (4.59 ± 1.24 mm) had progressed significantly relative to the MME preoperatively (3.63 ± 1.01 mm, *p* < 0.001). The clinical scores at 1 year postoperatively were improved significantly relative to the scores preoperatively (*p* < 0.001). The postoperative quadriceps muscle strength was correlated with ΔMME (correlation coefficient = -0.398, *p* = 0.030), and the change in quadriceps muscle strength was correlated with the KOOS-Quality of Life (correlation coefficient = 0.430, *p* = 0.018). Multiple regression analysis showed that the postoperative quadriceps muscle strength had a significant effect on ΔMME even when the body mass index and time from injury to surgery were included.

**Conclusions:**

After MM posterior root repair, patients with greater quadriceps muscle strength showed less MME progression. In addition, patients with greater improvement in quadriceps muscle strength had better clinical scores; therefore, continued rehabilitation aimed at improving quadriceps muscle strength after MM posterior root repair is recommended.

**Level of evidence:**

IV

## Background

The posterior root of the medial meniscus (MM) is the connection between the meniscus and the tibia and plays an important role in load distribution at the meniscus. MM posterior root tear (PRT) causes loss of hoop function of the meniscus, increased loading on the medial compartment, and further cartilage damage [1, 2]. Historically, treatment of MMPRT has involved conservative treatment and meniscectomy [3, 4]. Recently, meniscal repair has become the treatment of choice for MMPRT. Although MM posterior root repair has been reported to yield good clinical scores, it does not completely prevent medial joint space narrowing or progression of MM extrusion (MME) [5]. It is also known that the change in MME (ΔMME) is correlated with the medial joint space narrowing progression [5]. Therefore, reducing ΔMME is one of the goals of MMPRT treatment.

Quadriceps muscle strength is one of the most common indicators of lower limb muscle strength. An association between quadriceps muscle strength and symptomatic radiological osteoarthritis has been reported in the general population [6, 7]. Quadriceps muscle strength is reportedly correlated with knee osteoarthritis grade, pain, and clinical scores [8]. Similar to the meniscus, the quadriceps muscle strength is responsible for maintaining stability and shock absorption in the knee joint [9, 10]. Decreased quadriceps muscle strength affects knee joint stability and increases loading on the tibiofemoral joint, which can lead to cartilage and meniscus damage [11]. The association between quadriceps muscle strength and osteoarthritis or clinical scores after meniscectomy and anterior cruciate ligament reconstruction has been previously reported [12, 13]. However, there are few studies examining the association between quadriceps muscle strength and osteoarthritis or clinical scores after MM posterior root repair.

This study aimed to assess quadriceps muscle strength after MM posterior root repair and determine its relationship with clinical scores and MME. Our hypothesis was that patients with greater quadriceps muscle strength at 1 year after MM posterior root repair would have better clinical scores and less MME progression.

## Methods

### Patients

The study was conducted in accordance with the principles of the Declaration of Helsinki and received the approval of our Institutional Review Board (Okayama University, No. 1857). Written informed consent was obtained from all patients prior to participation.

At our institution, the indications for MM posterior root repair are a tibiofemoral angle of less than 180°, Kellgren–Lawrence grade 0–2, and mild cartilage lesions (International Cartilage Repair Society grade I or II). There are no exclusion criteria for surgical indications based on height, weight, body mass index, or patient activity. In the case of MMPRT with complete tears, we recommend surgical treatment as early as possible for patients with symptomatic knee pain and significant disruption to daily life. Partial MMPRT should first be treated conservatively, followed up carefully, and surgical treatment should be considered if the pain persists or worsens.

**Fig. 1:**
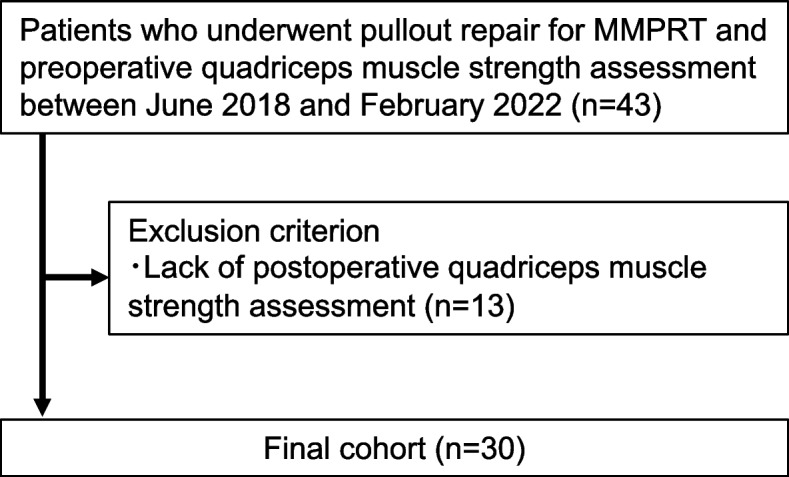
Study protocol flowchart. Abbreviation: MMPRT, medial meniscus posterior root tear

Forty-three patients who underwent preoperative quadriceps muscle strength measurements and pullout repair for MMPRT between June 2018 and February 2022 were initially included in the study. Although 1-year postoperative quadriceps muscle strength measurement was planned for the day before the second-look arthroscopy was performed, 13 patients were unable to perform the quadriceps muscle strength measurement owing to their hospitalization date or examination time. Finally, 30 patients were included in the study (Fig. 1). All patients underwent second-look arthroscopy at 1 year postoperatively.

Patient characteristics are summarized in Table 1. The MMPRT classification was made at the time of the initial repair surgery according to the report of LaPrade et al. [14].

**Table 1:** Patient characteristics

Characteristics	Value
Patients, n	30
Sex, male/female	10/20
Age, years	63.8 ± 8.8
[range]	[41–77]
Height, m	1.58 ± 0.07
[range]	[1.46–1.72]
Body weight, kg	66.4 ± 8.6
[range]	[54.0–79.0]
Body mass index, kg/m^2^	26.6 ± 3.2
[range]	[22.2–31.4]
Time from injury to surgery, days	146.9 ± 146.7
[range]	[19–644]
MMPRT classification, 1/2/3/4/5	4/24/0/2/0

Values are presented as means ± standard deviations or numbers

*MM* medial meniscus, *PRT* posterior root tear

### Surgical technique and rehabilitation protocol

**Fig. 2:**
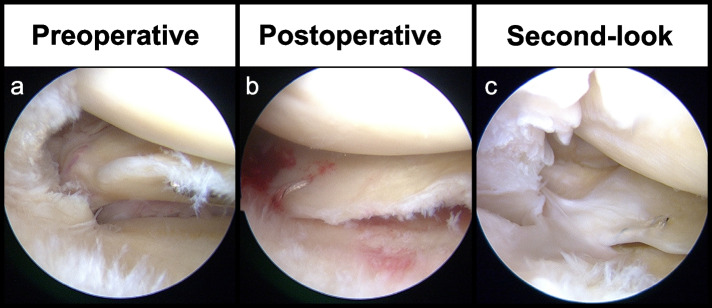
Preoperative, postoperative, and second-look arthroscopic findings (right knee). **a** Type 2 MMPRT, preoperatively. **b** MaxBraid sutures were applied using two cinch stitches. **c** Posterior root continuity and good synovial coverage were confirmed via second-look arthroscopy. Abbreviation: MMPRT, medial meniscus posterior root tear

Surgery was performed using transtibial pullout repair (Fig. 2). Sutures were applied to the posterior horn of the MM, threaded through the created tibial foramen, and fixed using a bioabsorbable screw. FiberStick (Arthrex, Naples, FL, USA), UltraTape (Smith & Nephew, Andover, MA, USA), and/or MaxBraid (Zimmer Biomet, Warsaw, IN, USA) sutures were used depending on the time of surgery. In addition, sutures were applied using two simple or two cinch stitches. Patients in whom sutures were added to the posteromedial portion of the posterior horn using an all-inside meniscal repair device were also included.

The rehabilitation protocol consisted of extension immobilization of the affected limb for the 1st postoperative week with no loading allowed. The range of motion of the knee joint increased to 30°, 60°, 90°, and 120° at 1, 2, 3, and 4 weeks postoperatively, respectively. The load was 20 kg at 1 week, 40 kg at 2 weeks, 60 kg at 3 weeks, and full at 4 weeks postoperatively. The rehabilitation period with a physical therapist was at least 3 months and included patellar setting and straight leg-raising training with a focus on quadriceps muscle training. Patients were instructed to continue similar training at home.

### Quadriceps muscle strength measurements

**Fig. 3:**
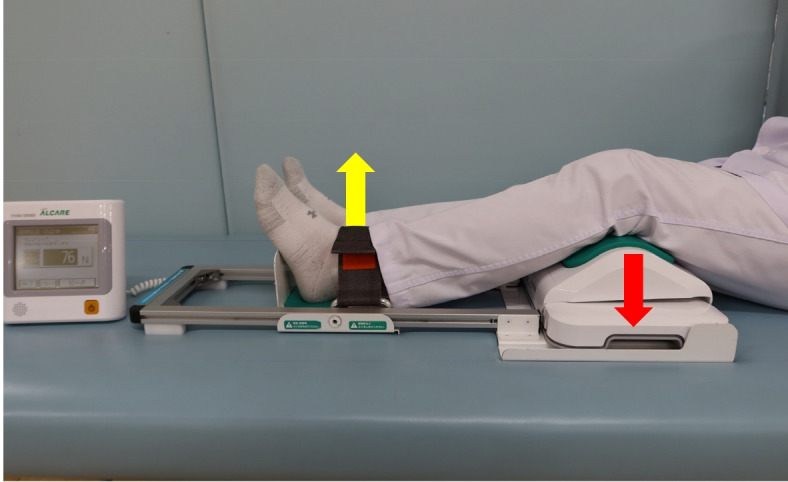
Measurement of quadriceps muscle strength. Quadriceps muscle strength was measured using the Locomo Scan-II instrument. Using the leverage principle, the knee is pushed into the measuring point (red arrow) by kicking up the belt that fixes the ankle joint (yellow arrow), allowing the measurement of knee extension strength, mainly of the quadriceps muscle strength

Quadriceps muscle strength measurements were taken the day before the pullout repair procedure and second-look arthroscopy. Quadriceps muscle strength was assessed by using the Locomo Scan-II device (ALCARE, Tokyo, Japan) and an assist frame specially designed for the Locomo Scan-II. The knee joint was fixed at 30° flexion and the ankle joint was fixed at 0°. Using the leverage principle, the knee was pushed into the measurement point by kicking the belt that fixed the ankle joint (Fig. 3). Measurements with this instrument were calculated in Newtons (N) and could be as low as 1 N and as high as 1,500 N.

### Magnetic resonance imaging assessments

**Fig. 4:**
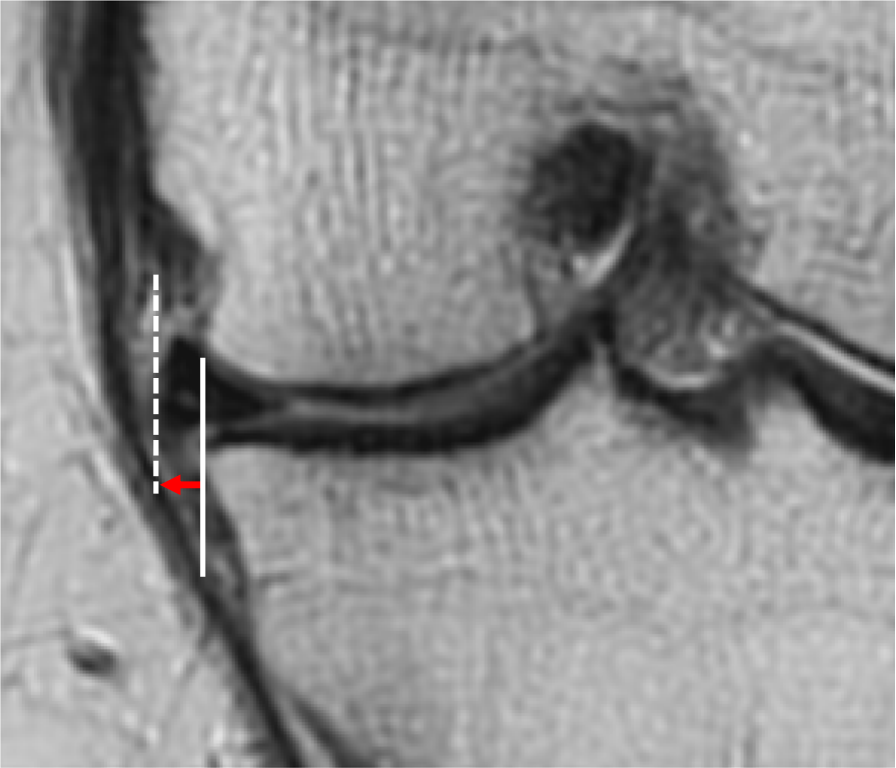
Measurement of MME. MME was measured by identifying the midpoint of the MM on a sagittal image and using a coronal image of that slice. The MME (red arrow) was defined as the distance from the medial margin of the tibia (white line), excluding osteophytes, to the medial edge of the MM (white dashed line). Abbreviations: MM, medial meniscus; MME, medial meniscus extrusion

Magnetic resonance imaging was undertaken both pre- and 1 year postoperatively to assess MME, which was measured by identifying the midpoint of the MM on the sagittal image and using a coronal image of that slice. MME was defined as the distance from the medial margin of the tibia to the medial edge of the MM (Fig. 4). Measurements were taken to the second decimal point.

### Clinical scores

Clinical scores were assessed preoperatively and at 1 year postoperatively. The evaluation was performed using the Knee Injury and Osteoarthritis Outcome Score (KOOS), International Knee Documentation Committee (IKDC) score, Lysholm score, Tegner score, and visual analog scale pain score. The KOOS has five scales: Pain, Symptoms, Activities of Daily Living (ADL), Sport and Recreation function (Sport/Rec), and Quality of Life (QOL).

### Statistical analysis

Measurements are expressed as mean values ± standard deviations. Statistical analyses were performed using the EZR software (Saitama Medical Center, Saitama, Japan). Wilcoxon’s signed-rank test was used to compare quadriceps muscle strength, MME, and clinical scores preoperatively and 1 year postoperatively. Pearson’s correlation coefficient (r) was used to evaluate the correlation of quadriceps muscle strength values with patient characteristics, clinical scores, and MME. Multiple regression analysis was performed to identify risk factors related to ΔMME. The patients were also divided into two groups based on the median postoperative quadriceps muscle strength and compared based on patient characteristics, preoperative quadriceps muscle strength, and ΔMME. Statistical significance was set at *p* < 0.05.

MME measurements were taken twice, 6 weeks apart, by two independent examiners. The inter- and intra-observer reliabilities of the measurements were determined with the intraclass correlation coefficient. Using post-hoc tests, we performed an actual power analysis to assess the preoperative and postoperative improvements in quadriceps muscle strength (G*Power, version 3.1.9.7; University of Düsseldorf, Düsseldorf, Germany).

## Results

Second-look arthroscopy confirmed the continuity of the posterior root in all patients.

The quadriceps muscle strength at 1 year postoperatively (355.1 ± 116.2 N) was significantly improved relative to that preoperatively (271.9 ± 97.4 N, *p* < 0.001) (Table 2). The MME at 1 year postoperatively (4.59 ± 1.24 mm) progressed significantly relative to that preoperatively (3.63 ± 1.01 mm, *p* < 0.001) (Table 2).

**Table 2:** Comparison of quadriceps muscle strength and MME preoperatively and at 1 year postoperatively

	Preoperative	Postoperative	*p*-value
Quadriceps muscle strength, N	271.9 ± 97.4	355.1 ± 116.2	< 0.001*
MME, mm	3.63 ± 1.01	4.59 ± 1.24	< 0.001*

Values are presented as means ± standard deviations

*p*-values were derived using Wilcoxon’s signed-rank test

*MME* medial meniscus extrusion

^*^Statistically significant

The clinical scores at 1 year postoperatively were significantly improved relative to the clinical scores preoperatively (all *p* < 0.001) (Table 3).

**Table 3:** Comparison of clinical scores preoperatively and at 1 year postoperatively

	Preoperative	Postoperative	*p*-value
IKDC score	44.3 ± 11.8	64.4 ± 16.3	< 0.001*
VAS pain score	38.9 ± 20.2	11.7 ± 17.0	< 0.001*
KOOS			
Pain	62.5 ± 13.2	85.0 ± 11.1	< 0.001*
Symptoms	64.3 ± 14.6	81.3 ± 10.9	< 0.001*
ADL	71.5 ± 14.0	86.2 ± 10.3	< 0.001*
Sport/Rec	24.8 ± 18.8	54.3 ± 24.3	< 0.001*
QOL	34.6 ± 18.5	57.6 ± 20.6	< 0.001*
Lysholm score	61.5 ± 15.0	86.7 ± 8.8	< 0.001*
Tegner score	2.3 ± 0.7	3.1 ± 0.7	< 0.001*

Values are presented as means ± standard deviations

*p*-values were derived using Wilcoxon’s signed-rank test

*ADL* activities of daily living, *IKDC* International Knee Documentation Committee, *KOOS* Knee Injury and Osteoarthritis Outcome Score, *QOL* Quality of Life, *Sport/Rec* Sport and Recreation function, *VAS* visual analog scale

^*^Statistically significant

**Fig. 5:**
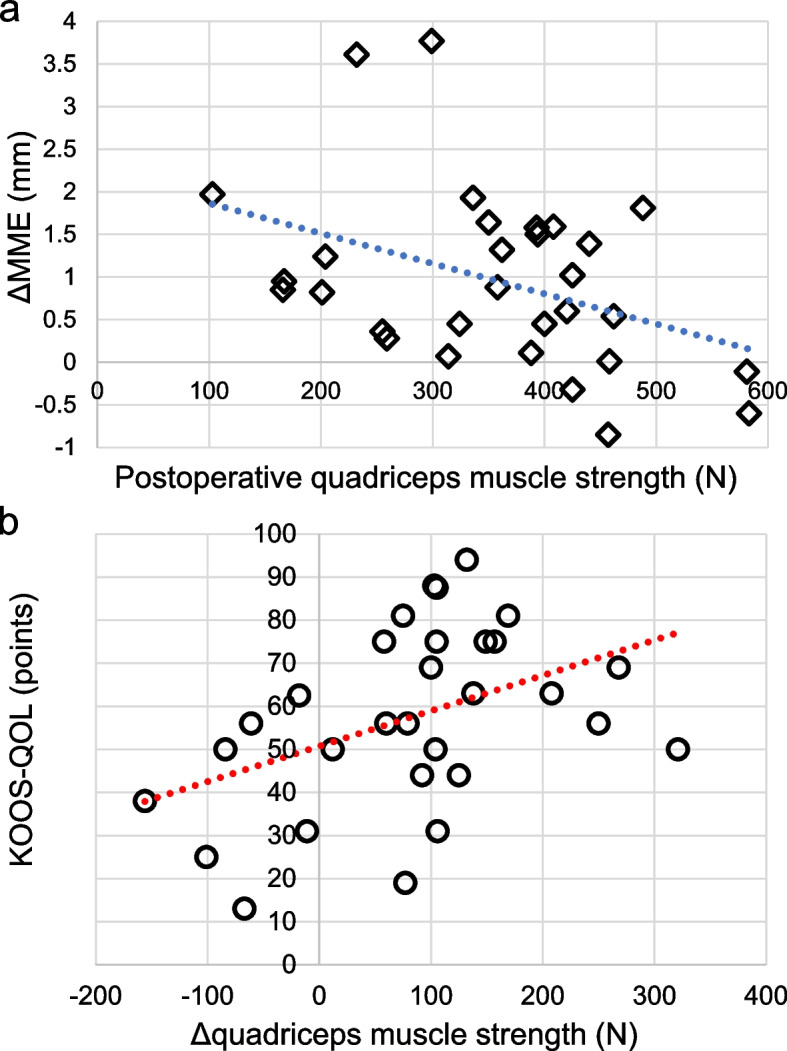
Scatter plots of the correlations. **a** The postoperative quadriceps muscle strength and ΔMME were negatively correlated (correlation coefficient = -0.398, *p* = 0.030). **b** Δquadriceps muscle strength and KOOS-QOL were positively correlated (correlation coefficient = 0.430, *p*-value = 0.018). Abbreviations: KOOS, Knee Injury and Osteoarthritis Outcome Score; QOL, Quality of Life; ΔMME, change in medial meniscus extrusion; Δquadriceps muscle strength, change in quadriceps muscle strength

The preoperative quadriceps muscle strength was correlated with body weight (*r* = 0.405, *p* = 0.027), IKDC score (*r* = 0.407, *p* = 0.026), KOOS-Pain (*r* = 0.398, *p* = 0.029), KOOS-ADL (*r* = 0.549, *p* = 0.002), KOOS-Sport/Rec (*r* = 0.491, *p* = 0.006), and preoperative Lysholm score (*r* = 0.486, *p* = 0.007; Table 4). The postoperative quadriceps muscle strength was correlated with the IKDC score (*r* = 0.419, *p* = 0.021), KOOS-Pain (*r* = 0.364, *p* = 0.048), KOOS-ADL (*r* = 0.444, *p* = 0.014), KOOS-Sport/Rec (*r* = 0.486, *p* = 0.007), KOOS-QOL (*r* = 0.572, *p* < 0.001), Tegner score (*r* = 0.680, *p* < 0.001), and ΔMME (*r* = -0.398, *p* = 0.030; Table 5, Fig. 5a). The change in quadriceps muscle strength (Δquadriceps muscle strength) was correlated with the KOOS-QOL (*r* = 0.430, *p* = 0.018; Table 6, Fig. 5b).

**Table 4:** Pearson’s correlation of preoperative quadriceps muscle strength

	Preoperative quadriceps muscle strength	
Variable	Correlation coefficient	*p*-value
Age, years	0.020	0.917
Height, m	0.245	0.192
Body weight, kg	0.405	0.027*
Body mass index, kg/m^2^	0.225	0.232
Time from injury to surgery, days	-0.179	0.344
Preoperative IKDC score	0.407	0.026*
Preoperative VAS pain score	-0.031	0.870
Preoperative KOOS-Pain	0.398	0.029*
Preoperative KOOS-Symptoms	0.121	0.523
Preoperative KOOS-ADL	0.549	0.002*
Preoperative KOOS-Sport/Rec	0.491	0.006*
Preoperative KOOS-QOL	0.361	0.050
Preoperative Lysholm score	0.486	0.007*
Preoperative Tegner score	0.298	0.109
Preoperative MME, mm	0.093	0.624
ΔMME, mm	-0.214	0.255

*ADL* activities of daily living, *IKDC* International Knee Documentation Committee, *KOOS* Knee Injury and Osteoarthritis Outcome Scor, *MME* medial meniscus extrusion, *QOL* Quality of Life, *Sport/Rec* Sports and Recreation function, *VAS* visual analog scale, *ΔMME* change in medial meniscus extrusion

^*^Statistically significant

**Table 5:** Pearson’s correlation of postoperative quadriceps muscle strength

	Postoperative quadriceps muscle strength	
Variable	Correlation coefficient	*p*-value
Age, years	-0.107	0.574
Height, m	0.270	0.149
Body weight, kg	0.265	0.157
Body mass index, kg/m^2^	0.058	0.762
Time from injury to surgery, days	-0.162	0.391
Postoperative IKDC score	0.419	0.021*
Postoperative VAS pain score	-0.222	0.238
Postoperative KOOS-Pain	0.364	0.048*
Postoperative KOOS-Symptoms	0.360	0.051
Postoperative KOOS-ADL	0.444	0.014*
Postoperative KOOS-Sport/Rec	0.486	0.007*
Postoperative KOOS-QOL	0.572	< 0.001*
Postoperative Lysholm score	0.325	0.080
Postoperative Tegner score	0.680	< 0.001*
Postoperative MME, mm	-0.156	0.412
ΔMME, mm	-0.398	0.030*

*ADL* activities of daily living, *IKDC* International Knee Documentation Committee, *KOOS* Knee Injury and Osteoarthritis Outcome Score, *MME* medial meniscus extrusion, *QOL* Quality of Life, *Sport/Rec* Sports and Recreation function, *VAS* visual analog scale, *ΔMME* change in medial meniscus extrusion

^*^Statistically significant

**Table 6:** Pearson’s correlation of Δquadriceps muscle strength

	Δquadriceps muscle strength	
Variable	Correlation coefficient	*p*-value
Age, years	-0.133	0.483
Height, m	0.070	0.715
Body weight, kg	-0.080	0.676
Body mass index, kg/m^2^	-0.141	0.456
Time from injury to surgery, days	-0.013	0.944
Postoperative IKDC score	0.326	0.078
Postoperative VAS pain score	-0.162	0.392
Postoperative KOOS-Pain	0.333	0.072
Postoperative KOOS-Symptoms	0.279	0.136
Postoperative KOOS-ADL	0.214	0.256
Postoperative KOOS-Sport/Rec	0.125	0.511
Postoperative KOOS-QOL	0.430	0.018*
Postoperative Lysholm score	0.304	0.102
Postoperative Tegner score	0.247	0.189
Postoperative MME, mm	-0.075	0.695
ΔMME, mm	-0.235	0.211

*ADL* activities of daily living, *IKDC* International Knee Documentation Committee, *KOOS* Knee Injury and Osteoarthritis Outcome Score, *MME* medial meniscus extrusion, *QOL* Quality of Life, *Sport/Rec* Sport and Recreation function, *VAS* visual analog scale, *ΔMME* change in medial meniscus extrusion, *Δquadriceps muscle strength* change in quadriceps muscle strength

^*^Statistically significant

Multiple regression analysis showed that the postoperative quadriceps muscle strength had a significant effect on ΔMME even when the body mass index and time from injury to surgery were included (Table 7).

**Table 7:** Multiple regression analysis for ΔMME

Variable	Coefficient	Std. Error	t-value	*p*-value
Constant	2.787	1.697	1.642	0.113
Body mass index, kg/m^2^	-0.019	0.059	-0.317	0.753
Time from injury to surgery, days	-0.000	0.001	-0.286	0.777
Postoperative quadriceps muscle strength, N	-0.004	0.002	-2.215	0.036*

*ΔMME* change in medial meniscus extrusion

^*^Statistically significant

The median postoperative quadriceps muscle strength was 375 N, dividing the study population into two groups with high (≥ 375 N) and low (< 375 N) postoperative quadriceps muscle strength. There were no obvious significant differences in patient characteristics, and the preoperative quadriceps muscle strength was significantly different between the two groups (*p* = 0.009; Table 8).

**Table 8:** Comparison of high and low postoperative quadriceps muscle strength groups

	Low postoperative quadriceps muscle strength (< 375 N)	High postoperative quadriceps muscle strength (≥ 375 N)	*p*-value
Patients, *n*	15	15	
Sex, male/female	3/12	7/8	0.245
Age, years	64.9 ± 8.8	62.6 ± 8.6	0.662
[range]	[50–77]	[41–77]	
Height, m	1.57 ± 0.07	1.59 ± 0.08	0.480
[range]	[1.50–1.72]	[1.46–1.71]	
Body weight, kg	65.2 ± 7.3	67.5 ± 9.7	0.618
[range]	[54.0–77.2]	[54.0–79.0]	
Body mass index, kg/m^2^	26.5 ± 3.6	26.6 ± 2.5	0.934
[range]	[22.2–31.4]	[22.6–30.1]	
Time from injury to surgery, days	144.0 ± 149.9	149.7 ± 143.4	0.803
[range]	[23–644]	[19–622]	
Preoperative quadriceps muscle strength, N	226.8 ± 76.4	317.0 ± 95.2	0.009*
[range]	[100–375]	[126–541]	
ΔMME, mm	1.34 ± 1.07	0.58 ± 0.84	0.093
[range]	[0.28–3.77]	[-0.85–1.81]	

Values are presented as means ± standard deviations or numbers

*p*-values were derived using Wilcoxon’s signed-rank test or Fisher’s exact test

*ΔMME* change in medial meniscus extrusion

^*^Statistically significant

The intra-observer reliabilities were 0.925 and 0.948, respectively, and the inter-observer reliability was 0.923 using the intraclass correlation coefficient. In the post-hoc analysis, the actual power to assess pre- and postoperative improvement in quadriceps muscle strength was 98.1% with a critical *p*-value of 0.05.

## Discussion

In this work, we assessed quadriceps muscle strength after MM posterior root repair and determined its relationship with clinical scores and MME. The most important findings of the present study were that patients with greater postoperative quadriceps muscle strength had less MME progression and that those with greater quadriceps muscle strength improvement had better clinical scores.

One of the strengths of our study is that second-look arthroscopy was performed in all cases, confirming the continuity of the posterior root repair. However, in many cases, MME progression was observed in the 1st postoperative year. In this study, we paid particular attention to the quadriceps muscle strength.

In this study, the Locomo Scan-II instrument was used to measure quadriceps muscle strength. Quadriceps muscle strength assessment using the Locomo Scan device is reportedly correlated with, and as useful as, assessment using the isokinetic machine [15]. The mean value for women in their 60 s using the Locomo Scan was 405 N [15]. In the present study, pullout repair for MMPRT and professional rehabilitation for at least 3 months postoperatively significantly improved quadriceps muscle strength at 1 year postoperatively. However, with an average age of 63.8 years, the mean quadriceps muscle strength in our study was 355.1 N at 1 year postoperatively, which was lower than the abovementioned average value for women in their 60 s. Hence, the quadriceps muscle strength of our patients had not fully recovered at 1 year after MM posterior root repair.

In the current study, there was a significant correlation between the preoperative quadriceps muscle strength and the preoperative clinical scores as well as between the postoperative quadriceps muscle strength and the postoperative clinical scores, indicating the importance of muscle strength. In addition, patients with improved quadriceps muscle strength showed better improvements in KOOS-QOL. The increase in activity owing to improved QOL creates a virtuous circle that leads to further improvements in muscle strength, which in turn leads to further improvements in QOL. Continuous rehabilitation is important for improving and increasing muscle strength. Quantitative assessment of quadriceps muscle strength provides information on current muscle strength, that is, the patient’s condition. Moreover, the patient’s knowledge and awareness of their improvements in muscle strength can provide further motivation for rehabilitation.

Herein, patients with greater postoperative quadriceps muscle strength tended to have a smaller ΔMME. In regard to ΔMME, the results showed that the absolute value of quadriceps muscle strength was more important than the amount of improvement in quadriceps muscle strength. Consistent with previous reports showing a relationship between muscle strength and knee joint stability [9, 10], patients with greater postoperative quadriceps muscle strength after MM posterior root repair may have more stable knee joints and less medial compartment stress. It is also known that ΔMME correlates with medial joint space narrowing progression [5]. ΔMME suppression is important to reduce the progression of arthropathic changes. Patient characteristics that have been reported to be related to ΔMME include body mass index [16] and time from injury to surgery [17]. Multiple regression analysis was used to examine the relationship between these characteristics and the postoperative quadriceps muscle strength; the postoperative quadriceps muscle strength had a significant effect on ΔMME even when the body mass index and time from injury to surgery were included. However, even in patients with greater quadriceps muscle strength, some patients showed an MME progression of approximately 2 mm, suggesting the involvement of various factors. Thus, there is a need to increase the number of patients and conduct various sub-analyses.

There is still no consensus regarding the timing of postoperative rehabilitation loading and range of motion training. Systematic reviews have reported that most studies have shown 2–3 weeks of knee joint immobilization in the extended position, with full weight-bearing at 6–8 weeks [18]. Our rehabilitation protocol, although gradual, was earlier than those in previous reports, with earlier timing of knee range of motion training and full weight-bearing at 4 weeks. Although the early rehabilitation protocol is desirable for improving and increasing muscle strength, it places a greater load on the posterior root repair area and may contribute to an increase in ΔMME. Moreover, the degree of postoperative rehabilitation intervention varied among the patients. After MM posterior root repair, better clinical outcomes have been demonstrated with professional rehabilitation than with home-based self-rehabilitation [19]. In this study, all patients received at least 3 months of rehabilitation by a physical therapist, but the presence or absence of rehabilitation after 3 months may have affected the pain and clinical scores.

Some biomechanical studies on the biomechanical changes in the knee joint after root repair have shown that root repair does not completely restore knee joint function [20]. Furthermore, biomechanical studies on the loosening of posterior root repair have reported that even if continuity of the posterior root is achieved, knee joint function is impaired depending on the degree of loosening of the repair [21]. The effects on ΔMME and muscle strength with respect to meniscus loosening should also be studied in the future.

This study has some limitations. First, this study had a relatively small sample size. Second, it was a retrospective study. Third, the surgical technique and instrumentation varied with the time of surgery. Different surgical procedures may have had an effect on pain and clinical scores. Fourth, we were unable to measure Δquadriceps muscle strength at 3 months or 6 months postoperatively. It is possible that the early postoperative quadriceps muscle strength recovery trend may be more related to MME and other factors. Fifth, this study could not include the contralateral quadriceps muscle strength. It is possible that the contralateral quadriceps muscle strength may have influenced the ΔMME and clinical scores.

## Conclusions

After MM posterior root repair, patients with greater quadriceps muscle strength had smaller ΔMME progression. In addition, patients with greater improvement in quadriceps muscle strength had better clinical scores; therefore, continued rehabilitation aimed at improving quadriceps muscle strength after MM posterior root repair is recommended.

## Data Availability

The datasets used and/or analyzed during the current study are available from the corresponding author on reasonable request.

## Abbreviations

ADL: Activities of daily living
IKDC: International Knee Documentation Committee
KOOS: Knee Injury and Osteoarthritis Outcome Score
MM: Medial meniscus
MME: Medial meniscus extrusion
PRT: Posterior root tear
QOL: Quality of Life
r: Pearson’s correlation coefficient
Sport/Rec: Sport and Recreation function
ΔMME: Change in medial meniscus extrusion
Δquadriceps muscle strength: Change in quadriceps muscle strength
